# Resting state brain activity in patients with migraine: a magnetoencephalography study

**DOI:** 10.1186/s10194-015-0525-5

**Published:** 2015-05-07

**Authors:** Hongxing Liu, Huaiting Ge, Jing Xiang, Ailiang Miao, Lu Tang, Ting Wu, Qiqi Chen, Lu Yang, Xiaoshan Wang

**Affiliations:** The Department of Neurology, Nanjing Brain Hospital, Nanjing Medical University, Guang Zhou Road 264, Nanjing, Jiangsu 210029 China; The MEG Center, Division of Neurology, Cincinnati Children’s Hospital Medical Center, 3333 Burnet Avenue, Cincinnati, OH 45220 USA; The MEG Center, Nanjing Brain Hospital, Nanjing, Jiangsu China

**Keywords:** Migraine, Magnetoencephalography (MEG), Mufti-frequency, Ictal activity

## Abstract

**Background:**

Recent advances in migraine research have shown that the cerebral cortex serves a primary role in the pathogenesis of migraine. Since aberrant brain activity in migraine can be noninvasively detected with magnetoencephalography (MEG), The object of this study was to investigate the resting state cortical activity differences between migraineurs and controls and its related clinical characteristics.

**Methods:**

Twenty-two subjects with an acute migraine and twenty-two age- and gender-matched controls were studied using MEG. MEG recordings were recorded 120 seconds during the headache attack. Analyze MEG signals from low (1–4 Hz) to high (200–1000 Hz)-frequency ranges.

**Results:**

In comparison with the controls, brain activity in migraine subjects was significantly different from that of the controls both in two frequency ranges (55–90 Hz, p < 0.001) and (90–200 Hz, p < 0.004). But the power value showed no significantly differences between control and migraines in all frequency ranges (p > 0.05). All the clinical characteristics had no significant correlation with aberrant brain activity.

**Conclusions:**

The results demonstrated that migraine subjects in resting state had significantly aberrant ictal brain activity that can be measured with neuromagnetic imaging techniques. The findings may facilitate the development of new therapeutic strategies in migraine treatment via alterations in cortical excitability with TMS and other medications in the future.

## Background

Migraine is a common episodic neurological disorder with a complex pathophysiology that manifests as recurrent attacks of headache that are typically throbbing and unilateral and often severe, with associated features such as hypersensitivity to multiple stimuli, including visual (photophobia), auditory (phonophobia), and sensory (cutaneous allodynia) stimuli during migraine attacks [1-5].

Migraine was originally speculated to be a disease solely involving the cranial vascular structures [1,6]. Recent studies have found that abnormal cortical excitability, believed to be due to calcium channelopathy, appears to play an important role in predisposing to spontaneous, cortical spreading depression, which is suggested to be one basis for the pathophysiology of migraine with aura [7]. Abnormal activation in the contralateral visual cortex in migraine with aura has been observed using functional magnetic resonance imaging (fMRI) [8]. The nature and mechanisms of the primary brain dysfunction(s) leading to the onset of a migraine attack, susceptibility to cortical spreading depression, and episodic activation of the trigeminovascular pain pathway remain largely unknown and are major outstanding issues that need to be addressed to further understand the neurobiology of migraine [2].

Magnetoencephalography (MEG), a relatively new clinical neuroimaging modality, is well suited for the study of cortical dysfunction in migraine because it can noninvasively detect and localize neuromagnetic signals associated with functional brain activation [9,10]. MEG is considered to be superior to scalp-electroencephalography (EEG) because the skull, skin, and other tissues can distort electric signals while magnetic signals can pass through these tissues without significant distortion [11,12]. Previous MEG studies investigating migraine have typically focused on neuromagnetic waveforms in a low-frequency range, such as DC-MEG signals. It has been shown that neuromagnetic brain activity is significantly increased in patients with migraine. Other studies have documented the hyperexcitability of the occipital cortex to visual stimulation in patients with migraine using MEG [13-15]. Another series of recent studies involving patients having an acute migraine or an acute exacerbation of chronic migraine showed that there were significant delays in both auditory processing of information and in motor response to cues, compared with age- and gender-matched controls [16-20]. The excitability of cell membranes appears to be a fundamental factor in the brain’s susceptibility to migraine attack [21].

The aim of the present study was to quantify the spatial and spectral abnormalities in brain activity at a resting state with MEG and to assess its relationship with clinical characteristics. To address this aim, we analyzed MEG signals from low- to high-frequency ranges. To the best of our knowledge, the present study is the first to examine the neuromagnetic signatures of aberrant resting-state brain activity in patients with acute migraine during headache attacks. With a better understanding of the cerebral mechanisms of migraine, headache treatments targeting at cortical dysfunctions (for example, transcranial magnetic stimulation, which currently shows great promise) could be refined and their clinical usefulness can be significantly improved.

## Methods

### Subjects

Twenty-two subjects with acute migraine (all women; mean age 31.4 years; standard deviation 3.6 years; age range 20 to 40 years) were recruited from the Department of Neurology, Nanjing Brain Hospital Affiliated Nanjing Medical University. The participants were pre-screened by neurologists specialized in headache at the authors’ Headache Clinic at Nanjing Brain Hospital. If a participant met the criteria and was interested in the MEG study, a researcher would explain the research protocol and obtain written informed assent and consent forms from the participants. The Institutional Review Board at Nanjing Brain Hospital formally approved the research protocol, assent, and consent forms. Inclusion criteria were: 1) migraine with or without aura as defined in the International Classification of Headache Disorders, 2nd Edition [18,22]; and 2) no other neurological disorder. Controls were recruited to match the migraine subjects for age and gender (Table 1), and met the inclusion criteria of 1) healthy, without a history of neurological disorder, headache, or brain injury; and 2) age-appropriate hearing, vision, and hand movement. Exclusion criteria for all participants were: 1) presence of an implant, such as cochlear implant devices, a pacemaker, or neurostimulator; devices containing electrical circuitry, generating magnetic signals, or other metal devices that could produce visible magnetic noise in MEG data; and 2) noticeable anxiety (expressing worry about the tests with noticeable physical trembling or sweating) and/or inability to readily communicate with the personnel operating the MEG equipment; and 3) taking antiepileptic drugs in the previous 3 days [14,20].

**Table 1:** **Controls characteristics**

**Control**	**Sex**	**Age (years)**	**Menstruation**
1	F	21	No
2	F	35	Yes
3	F	33	No
4	F	32	Yes
5	F	23	Yes
6	F	29	Yes
7	F	27	No
8	F	36	Yes
9	F	20	No
10	F	37	No
11	F	32	No
12	F	40	No
13	F	30	No
14	F	35	Yes
15	F	38	No
16	F	33	No
17	F	34	No
18	F	20	No
19	F	36	Yes
20	F	35	No
21	F	27	No
22	F	37	No

### MEG recording

The brain signals of each subject were recorded for 2 min. The MEG data were recorded using a CTF 275-Channel MEG system (VSM Medical Technology Company, Canada) that assessed the entire head in a magnetically shielded room in the MEG Center at Nanjing Brain Hospital. Before data acquisition commenced, all subjects were asked to remove all possible metals from their body. Subsequently, electromagnetic coils were attached to the nasion, left and right pre-auricular points of the subject. These three coils were subsequently activated at different frequencies to measure subjects’ head positions relative to the MEG sensors. During the MEG recordings, the subjects were required simply to keep still as much as possible, close their eyes and not to think of anything systematically. The subjects were also asked to lie still comfortably in a supine position and avoid swallowing or clenching their teeth during the entire procedure.

To ensure all the subjects were awake, we analyzed Alpha waves. Alpha (8 Hz to 12 Hz) waves are one type of brain waves and predominantly originate from the occipital lobe during wakeful relaxation with closed eyes. Alpha waves are reduced with open eyes, drowsiness and sleep. In our study, the main activity of 8 Hz to 12 Hz (α wave) neuromagnetic regions in all of the subjects and controls were located in the occipital lobe.

The sampling rate of the MEG recordings was 4000 Hz per channel. The subject’s head positions relative to the MEG sensors were measured using the three coils. The head positions were measured at the beginning and the end of procedures. If head movement during a recording was >5 mm, the set was considered to be “bad” and an additional trial was recorded. The MEG signals were acquired with noise cancelation of third-order gradients.

### Magnetic Resonance Imaging (MRI) scan

MRI scanning was performed on all subjects using a 1.5 T scanner (Sigma, GE, USA). Three MRI marks were placed in locations corresponding to the locations of the three coils used in the MEG recordings to co-register the MRI and MEG data. The scan field of view was 240 mm, field of view phase was 100%, repetition time was 6600 ms, echo time was 93 ms, and slice thickness was 1.00 mm.

### MEGData analysis

MEG waveforms were visually inspected for identifying magnetic noise and artifacts. Any MEG data with noticeable magnetic noise or artifact (>6 pT) were excluded from the analyses. MEG data without noise or artifact were considered to be “clean MEG data.” MEG signals in delta (1 Hz to 4 Hz), theta (4 Hz to 8 Hz), alpha (8 Hz to 12 Hz), beta (12 Hz to 30 Hz), low-gamma (30 Hz to 45 Hz), high-gamma (55 Hz to 90 Hz), ripple (90 Hz to 200 Hz), and high-frequency oscillations (HFOs) (200 Hz to 1,000 Hz) were analyzed. The selection of bandwidth for ripple and HFO frequency bands was mainly based on previous studies investigating high-frequency brain signals in epilepsy [23,24]. Neuromagnetic sources were localized with accumulated source imaging [25,26]. Accumulated source imaging was defined as the volumetric summation of source activity over a period of time, which was specifically developed and optimized to analyze ictal activity in migraine patients. The source localization algorithms of accumulated source imaging were based on recent reports on MEG source reconstruction [27]. Accumulated source imaging could localize correlated sources by using node-beam lead fields [25]. Since each node-beam lead field represented a form of “source-beamformer” or “sub-space solution”, accumulated source imaging had multiple source beamformer to separate correlated sources. The detailed mathematical algorithms and validations are described in a recently published article [25].

**Figure 1:**
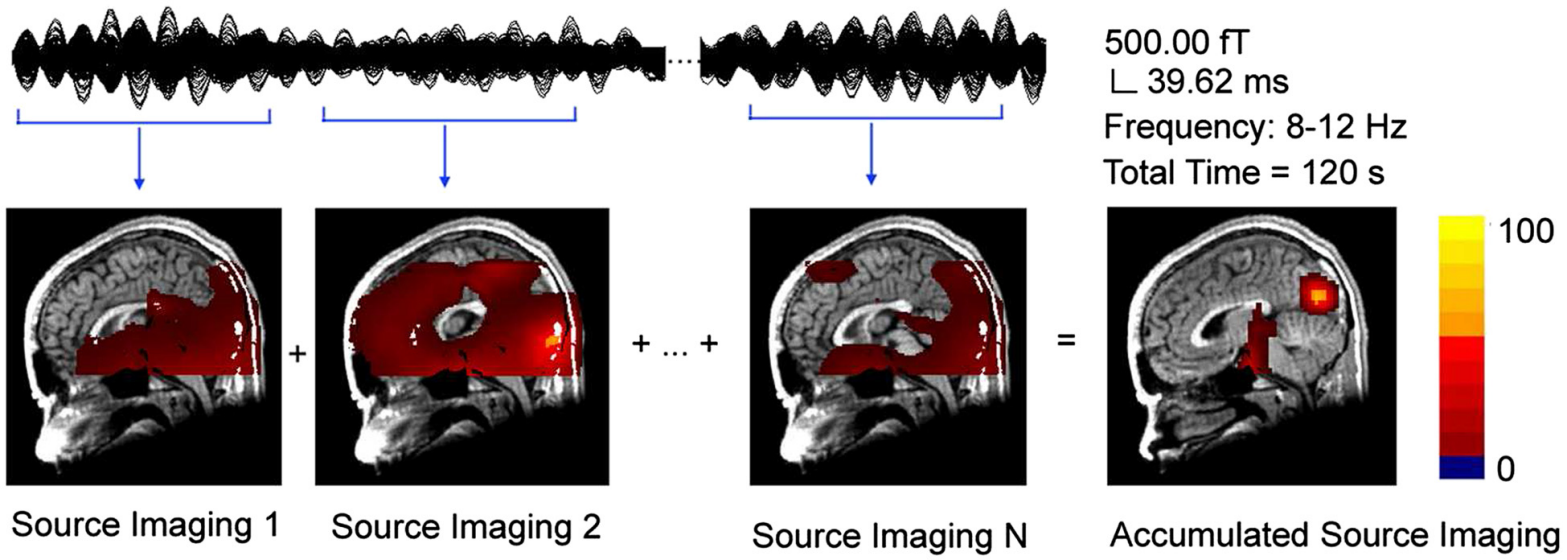
Illustration showing the principle of accumulated source imaging. To quantify magnetic sources for a 2-minute recording, a slide-time-window is used to compute a set of volumetric source data for the entire dataset. An accumulated source image is produced by spatially adding all source data together. In this example, MEG data in 8–12 Hz (alpha activity) are localized to the occipital cortex by accumulated source imaging.

To provide an accurate description of brain activity, each voxel in source imaging had at least three parameters: (1) strength of source activity, (2) correlation coefficients between the magnetic signals detected by the MEG system and the magnetic signals generated by the sources, and (3) the statistical significance of the differences between the magnetic signals detected by MEG system and the magnetic signals generated by the sources. We quantified source location with X, Y and Z axes in a 3D coordinate system defined by three fiducial points in MEG. The measurements of neuromagnetic source strength were performed using MEG Processor, a software package implementing the new method of accumulated source imaging [25]. See in Figure 1.

### Statistical analyses

The odds ratio of activity in brain areas among the migraine and control groups was analyzed with Fisher’s exact tests. Analysis of variance (ANOVA) analysis was applied to the power value difference between migraine subjects versus controls. The correlation between migraine clinical characteristics (age, headache history, duration, pain type, frequency, and menstruation) and MEG parameters (source location and magnetic source power) were analyzed using Spearman correlation coefficients. For multiple comparisons, a Bonferroni multiple comparisons correction was used. Significance was accepted at the level of p < 0.05 for a single test. If multiple testing were to be taken into account, the significance level for each of these tests was reduced from 0.05 to 0.025 (two parameters) or 0.016 (three parameters).

## Results

### Clinical characteristics

Of the 22 subjects, six (27.27%) had migraine with aura and 16 (72.73%) had migraine without aura. None of the subjects with migraine with aura experienced the aura during the MEG recording. The average age was 31.63 ± 3.61 years. The headaches of seven (31.81%) women were related to menstruation. Fourteen of the 22 subjects had bilateral headaches (63.64%). The clinical characteristics are presented in Table 2.

**Table 2:** **Patients clinical characteristics**

**Patients**	**Sex**	**Age (years)**	**History (years)**	**Attack Frequency**	**Menstruation**	**Duration**	**Pain**	**Pain Location**	**Pain Intensity**	**Photophobia**	**Phonophobia**	**Nausea**	**Onset to scan**
						**(hours)**	**Type**						**(hours)**
1	F	21	7	1-2/ month	No	12	Pressure	Bilateral	Moderate	No	No	Yes	4.5
2	F	35	8	1/month	Yes	3	Pressure	Bilateral	Severe	Yes	Yes	Yes	2
3	F	33	6	2/ month	No	14	Pressure	Unilateral	Severe	Yes	Yes	No	5.5
4	F	32	9	1/month	Yes	12	Throbbing	Bilateral	Moderate	Yes	Yes	Yes	4
5	F	23	10	1-2/ month	Yes	10	Pressure	Unilateral	Severe	No	No	Yes	3
6	F	29	8	1/month	Yes	12	Pressure	Bilateral	Severe	Yes	Yes	Yes	5
7	F	27	10	1-2/ month	No	14	Pressure	Both	Moderate	Yes	Yes	Yes	6
8	F	36	3	1/month	Yes	20	Constant	Bilateral	Severe	Yes	Yes	No	4.5
9	F	20	4	1-2/month	No	8	Constant	Bilateral	Moderate	No	No	Yes	4
10	F	37	5	1/month	No	6	Throbbing	Bilateral	Severe	Yes	Yes	Yes	2
11	F	32	7	1-2/month	No	10	Constant	Bilateral	Severe	No	No	Yes	3
12	F	40	10	2/week	No	24	Throbbing	Bilateral	Mild	Yes	Yes	No	6
13	F	30	5	1/month	No	8	Throbbing	Bilateral	Severe	Yes	Yes	Yes	4
14	F	35	10	2-3/month	Yes	24	Pressure	Unilateral	Mild	Yes	Yes	Yes	8
15	F	38	15	2-3/week	No	8	Constant	Bilateral	Severe	Yes	Yes	Yes	3
16	F	33	8	<1 /month	No	6	Throbbing	Both	Severe	Yes	Yes	No	3
17	F	34	15	1-2/week	No	15	Throbbing	Bilateral	Moderate	No	No	Yes	5
18	F	20	10	1/month	No	24	Throbbing	Unilateral	Moderate	Yes	Yes	No	7
19	F	36	10	<1/month	Yes	12	Constant	Bilateral	Mild	Yes	Yes	No	5
20	F	35	8	2/month	No	3	Pressure	Unilateral	Severe	Yes	Yes	Yes	1.5
21	F	27	3	1/month	No	4	Constant	Bilateral	Severe	Yes	Yes	Yes	3
22	F	37	5	<1/month	No	13	Throbbing	Unilateral	Moderate	Yes	Yes	No	5

### Source location

The accumulated magnetic source imaging (MSI) showed that the main activity of neuromagnetic region of resting state in all the eight frequency ranges (Table 3).

**Table 3:** **Source location**

1-4hz	L	DBA	CC	30-45hz	L	DBA	CC
	N				N		
	G				G		
	Migraine	22	0		Migraine	19	3
	Control	22	0		Control	22	0
4-8hz	L	DBA	OC	55-90hz	L	DBA	LFC
	N				N		
	G				G		
	Migraine	2	20		Migraine	11	11
	Control	1	21		Control	21	1
8-12hz	L	DBA	OC	90-200hz	L	DBA	LFC
	N				N		
	G				G		
	Migraine	22	22		Migraine	13	9
	Control	22	22		Control	21	1
12-30hz	L	DBA	OC	0.2-1Khz	L	DBA	CC
	N				N		
	G				G		
	Migraine	4	18		Migraine	22	0
	Control	2	20		Control	22	0

DBA = deep brain area; CC = cerebral cortex; OC = occipital cortex; LFC = lateral frontal cortex; L = location; N = numbers; G = group.

**Figure 2:**
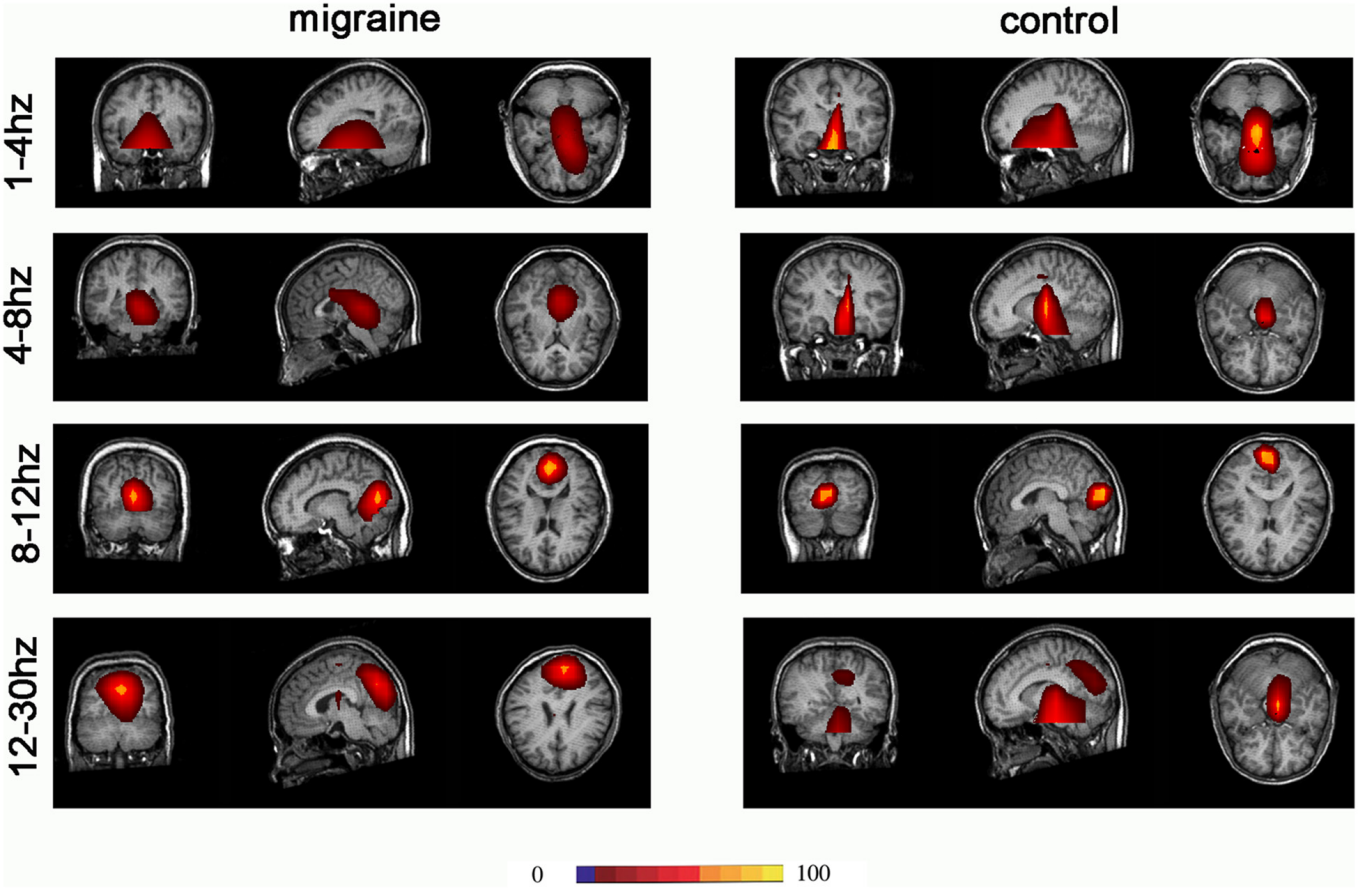
Magnetic source images showing intrinsic brain activity in 1–30 Hz frequency range in a subject with Migraine and a control. MEG data in 8–12 Hz (alpha activity) are localized to the occipital cortex in both of them.

In 8 Hz to 12 Hz (α wave), the main activity of neuromagnetic regions in almost all of the subjects and controls were located in the occipital lobe. There were no significant difference between migraine subjects and controls (Figure 2).

In some frequency ranges (1 Hz to 4 Hz, 4 Hz to 8 Hz, 12 Hz to 30 Hz, 30 Hz to 45 Hz, 200 Hz to 1000 Hz), the main activities of the neuromagnetic region in subjects with migraine were not significantly different compared with controls. Most were located in the deep brain areas, such as the brainstem, thalamus, and cerebellum, and were not analyzed in detail.

**Figure 3:**
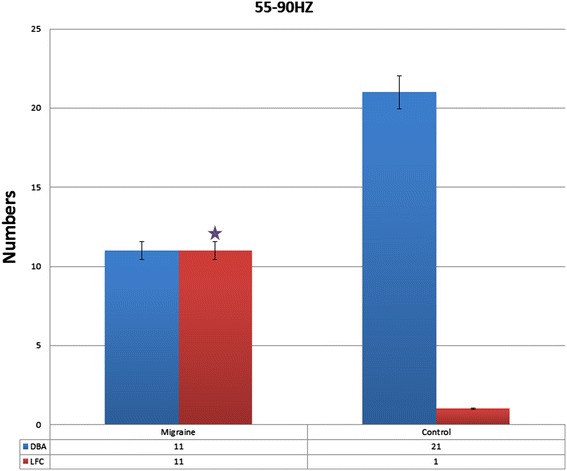
The number of migraine subjects and control in different neuromagnetic activity areas in 55–90 Hz, migraine subjects have significantly higher odds of activation in lateral frontal cortex the compared to control in resting state. DBA means deep brain area. *p* < .05.

In the frequency band of 55 Hz to 90 Hz, the MSI showed that the main neuromagnetic activity in the resting state exhibited some differences. Compared with the control, the main neuromagnetic activity in subjects with migraines was observed to be in a significantly different brain region in the 55 Hz to 90 Hz frequency range (p < 0.05; Table 1). In the majority of the controls (21 of 22) and some of the subjects with migraine (11 of 22), the main neuromagnetic activity was in the DBA. The main neuromagnetic activity was located in the lateral frontal cortex (LFC) in some of the subjects with migraine (11 of 22) and very few controls (one of 22) (Figure 3).

**Figure 4:**
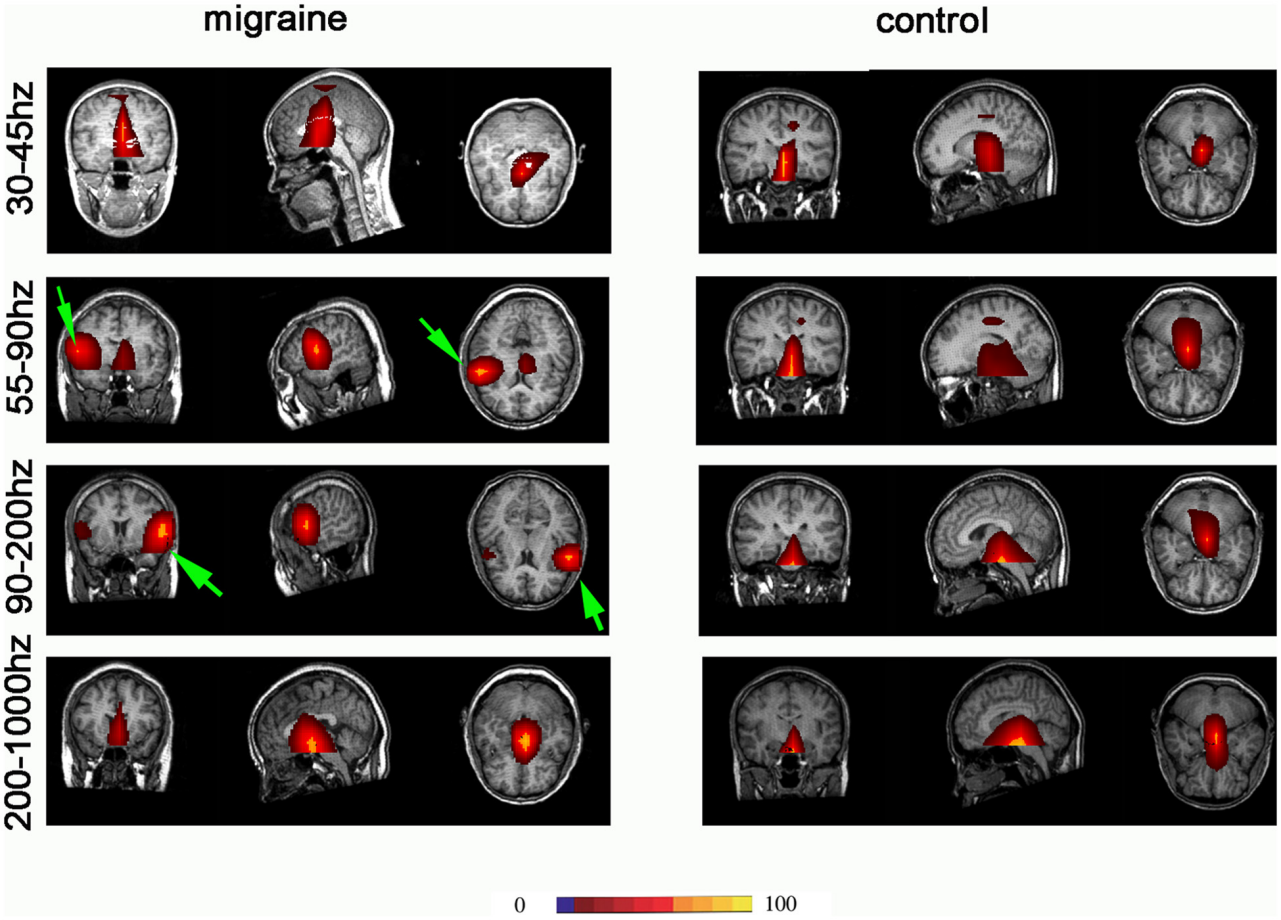
Magnetic source images showing intrinsic brain activity in 45–1000 Hz frequency range in a subject with migraine and a control. In comparison to the control, the subject with migraine shows significantly altered patterns of source imaging in 55–90 Hz and 90–200 Hz frequency ranges. A green arrow points to the region that shows significant differences between the subject with migraine and the control.

**Figure 5:**
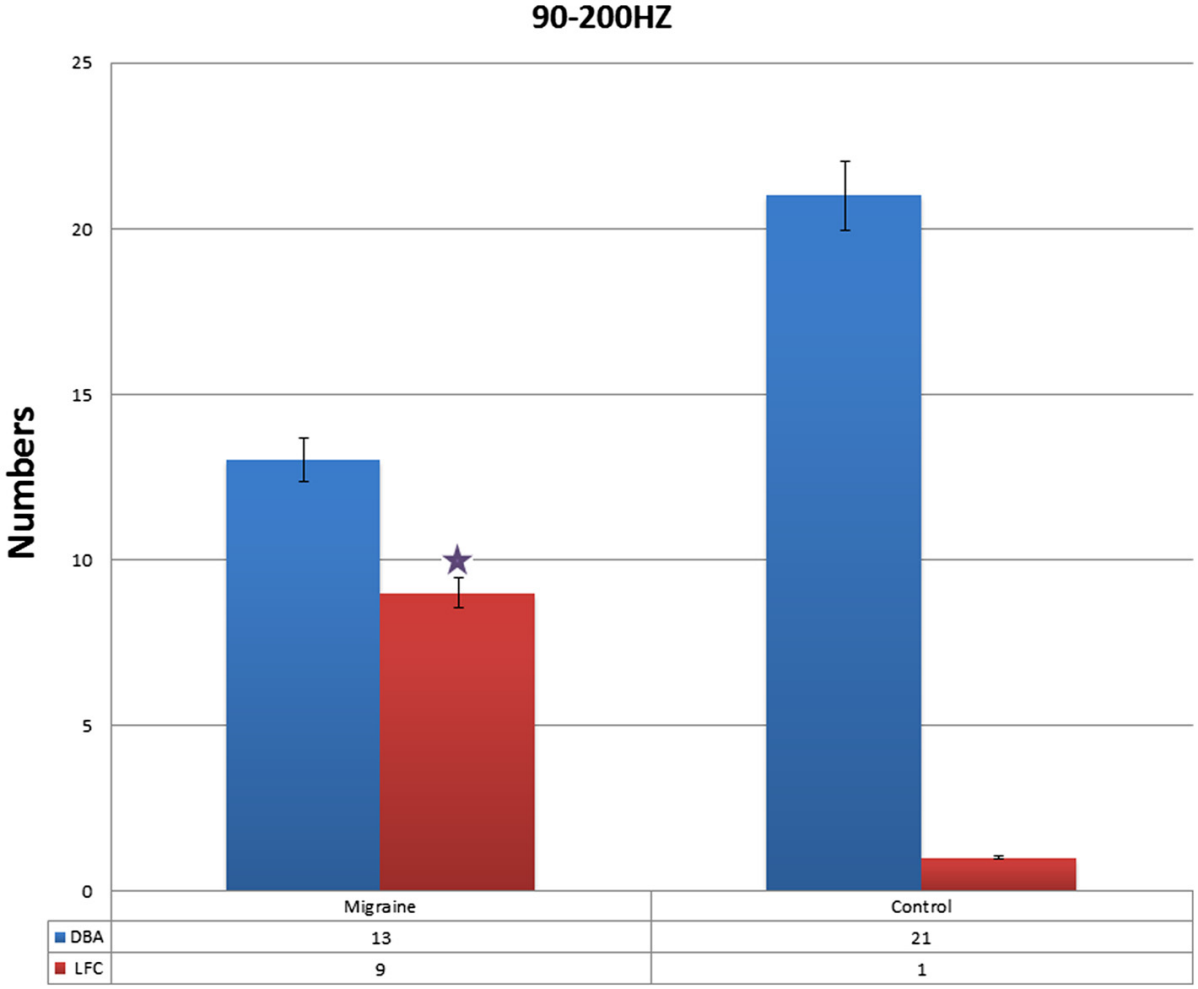
The number of migraine subjects and control in different neuromagnetic activity areas in 90–200 Hz, migraine subjects have significantly higher odds of activation in lateral frontal cortex the compared to control in resting state. DBA means deep brain area. *p* < .05.

In the frequency band of 90 Hz to 200 Hz, the result was similar to 55 Hz to 90 Hz. Compared with controls, the main neuromagnetic activity in subjects with migraines was also observed to be in a significantly different brain region in the 90 Hz to 200 Hz frequency range. In most of the controls (21 of 22) and some of the subjects with migraine (13 of 22), the main neuromagnetic activity was in the DBA. In some of the subjects with migraine (nine of 22) and very few controls (one of 22), the main neuromagnetic activity was located in the LFC [p = 0.004 (<0.05)] (Figures 4 and 5).

In all frequency rages, the main neuromagnetic activity was compared between subjects with migraine with aura and those with migraine without aura. There was no significant difference between these two groups (p > 0.05).

### Source strength

The distributions of source power can be displayed on the individual MRI images. There was no significant difference in resting-state main activity of the neuromagnetic source power between the groups with migraine compared with controls in all eight-frequency ranges (from 1 Hz to 4 Hz, to 200 Hz to 1000 Hz; p > 0.05). The source power was also compared between subjects with migraine with aura and subjects with migraine without aura. There was also no significant difference between these groups (p > 0.05) (Table 4).

**Table 4:** **Neuromagnetic source strength of intrinsic brain activity in subjects with migraine and controls**

**Frequency source**	**Migraine subject**	**Control**	**p value**
1–4 Hz	89.16 ± 12.64	95.64 ± 8.31	p > 0.05
4–8 Hz	82.06 ± 16.89	88.19 ± 10.08	p > 0.05
8–12 Hz	84.53 ± 12.47	95.07 ± 8.32	p > 0.05
12–30 Hz	70.65 ± 16.75	75.68 ± 9.31	p > 0.05
30–45 Hz	53.18 ± 17.13	55.19 ± 9.97	p > 0.05
55–90 Hz	65.45 ± 11.98	57.76 ± 9.18	p > 0.05
90–200Hz	43.52 ± 8.56	40.45 ± 7.51	p > 0.05
200–1000Hz	28.73 ± 4.32	26.75 ± 3.46	p > 0.05

### Clinical characteristics correlation

Results of correlation analyses demonstrated that there were no significant correlations between clinical characteristics (age, headache history, duration, pain type, frequency) and source location (p > 0.05). There were also no significant correlations between source power and clinical characteristics (p > 0.05). Because all subjects were women, the relationships between menstruation and both source location and source power were also analyzed; however, no significant correlations were observed (p > 0.05).

## Discussion

The present study investigated the differences in resting-state brain activity and its related clinical characteristics, both in source location and source level from low- (1 Hz to 4 Hz) to high- (200 Hz to 1000 Hz) frequency neuromagnetic signals using MEG between a group of patients with acute migraines and a group of control patients. The source location data revealed differences between the two groups in two frequency ranges: 55 Hz to 90 Hz, and 90 Hz to 200 Hz.

To analyze frequency-dependent changes, MEG signals were divided into eight ranges (1 Hz to 4 Hz, 4 Hz to 8 Hz, 8 Hz to 12 Hz, 12 Hz to 30 Hz, 30 Hz to 45 Hz, 55 Hz to 90 Hz, 90 Hz to 200 Hz, 200 Hz to 1000 Hz). MEG is sensitive to magnetic noise generated by power lines, which were tentatively avoided by analyzing signals in 30 Hz to 45 Hz and 55 Hz to 90 Hz because the power-line noise in China is 50 Hz. Accumulated source imaging is suitable for the study of resting-state brain activity. According to previous reports of functional mapping [16,28-30], if the signals were produced by noise, they should not be consistently localized to a brain area. Furthermore, similar to the conventional averaging for detecting time-locked brain activity in evoked potentials and accumulated spectrograms [24,31], accumulated source imaging detects spatial- and frequency-locked brain activity over a period of time. However, magnetic noise commonly occurs in random frequencies and locations, except power-line noise. To validate the method, we volumetrically localized alpha activity (8 Hz to 12 Hz) for all subjects. Promisingly, alpha activity was consistently localized to the occipital cortex in controls. The reliability of the new approach has been verified with functional mapping [22,32-36], intracranial electroencephalography data, and surgical outcomes [24,31,37], and we are confident that the results are reliable and reproducible.

Most previous studies investigating migraine using MEG have used stimulation, including visual, auditory and motor. This series of studies found many significant differences between subjects with acute migraine and control subjects. Even in the same patient, ictal and interictal measures are significantly different [14,17,18,20]. Previous MEG studies investigating resting-state migraine typically only analyze the waveform and amplitude, or focus on small frequency ranges. These studies have resulted in several observations, such as suppression of spontaneous cortical activity, long-duration field changes, and large-amplitude waves of several seconds’ duration [10]. In the present study, we used a relative new analytical method that enables more direct and accurate analyses. To our knowledge, the present study is the first MEG accumulated source imaging study to investigate resting-state migraine in such a large number of frequency bands in high-frequency ranges in addition to low-frequency ranges. The entire brain was analyzed and volumetric rendering technology was utilized, which enabled us to visualize the functional organization of intrinsic brain activity as volumetric source imaging. Compared with conventional analyses of brain waveforms, our new approach provides spatial, frequency, as well as volumetric descriptions of the abnormalities of brain activity in subjects with migraine compared with controls.

The results of the present study reveal that subjects with acute migraine have a high likelihood of neuromagnetic activity in LFC compared with controls in two frequency ranges. Lateral frontal cortex has a relevant role in modulating behavioral responses to aversive stimuli and may significantly influence pain experience [38]. Since migraine is associated with cortical hyperexcitability, resting-state abnormal activity may play a key role in the cascade of migraine attacks. Resting-state abnormal activity may also influence brain activity following other stimulation, including visual, auditory and motor. The activities during stimulation are all based on the resting state; it is possible that the aberrant resting-state brain activity can provide a better, direct explanation of the pathogenesis of migraine. This observation may be critical for developing spatially targeted treatments for migraine. For example, high-frequency repetitive transcranial magnetic stimulation increases and low-frequency repetitive transcranial magnetic stimulation decreases neural excitability of the stimulated cortex [39-41].

If MEG could reliably reveal the location and types of cortical dysfunction occurring during migraine attacks, all of the preventions and treatments targeted at cortical excitability [42-44] could be refined and optimized. Therefore, we consider the present study to lay an important foundation for the clinical management of migraine in the future.

Although our data showed differences in source location between migraine and control groups, the source power and related clinical characteristics were not significantly different. This is likely due to limited number of subject groups; only two groups were included (ictal migraine and controls), and it is more than likely that the two groups had individual variations. In future research, an interictal migraine group may be added. Comparing three groups may minimize individual differences. Another limitation of the current study is the number of the participants. This is a limitation common to many MEG studies of multi-frequency analysis if high-frequency brain activity is included, because the study of high-frequency brain activity requires a high sampling rate of MEG data, which produces an unusually high number of MEG data points. Consequently, considerable computing power and time are required to analyze these data points and establish the spatial and spectral characteristics for all frequency ranges for each subject. However, with the development of the computer technology, especially the graphics processing unit, the core unit of graphics card, can solve this problem with the advantages of parallel computing, high-speed floating-point performance and high memory bandwidth. In addition, some confounding factors, such as the use of clinical drugs, may affect the results. Therefore, further investigation is necessary.

## Conclusions

The results of the present study have demonstrated female patients with migraine during a headache attack had a higher likelihood of neuromagnetic activation in cerebral cortex. The findings support the notion that migraine is associated with cortical hyperexcitability. The abnormal ictal resting state brain activity may play a key role in the cascade of migraine attacks. These findings may be useful for developing spatially targeted treatment (such as rTMS) for migraine treatment in the future.
